# Disseminated Pulmonary Mycosis Caused by *Candida tropicalis* in an 11-Year-Old Male Patient with Chronic Granulomatous Disease

**DOI:** 10.1155/2022/7089907

**Published:** 2022-09-19

**Authors:** Ali Alsuheel Asseri, Ahmed Al-Jarie, Alshima Alassim, Mohamed E. Hamid, Hamza AlGhamdi

**Affiliations:** ^1^Department of Child Health, King Khalid University, Abha 62529, Saudi Arabia; ^2^Abha Maternity and Children Hospital, Abha, Saudi Arabia; ^3^College of Medicine, King Khalid University, Abha 62529, Saudi Arabia; ^4^Department of Microbiology, College of Medicine, King Khalid University, Abha, Saudi Arabia; ^5^Department of Pediatrics, King Fahad Medical City, Riyadh, Saudi Arabia

## Abstract

Invasive fungal infection is a major threat to chronic granulomatous disease (CGD) patients. We present a rare case of invasive mycosis in a CGD boy. An 11-year-old preadolescent boy presented with fever, hypoxia, and dyspnea. Physical examination revealed left neck enlarged lymph nodes with healed scars. The chest revealed bilateral diminished air entry with bilateral coarse crackles. Peripheral blood leukocyte count was 28.260/*μ*L with 84% neutrophil, 11% lymphocyte, and 4.4% monocyte. The patient's condition deteriorated regardless of the empirical antibacterial against MRSA and suspected tuberculosis. A sputum sample was submitted for mycological investigation, and budding yeasts with pseudohyphae were detected in the direct smear and were isolated in pure culture using Sabouraud agar. *Candida tropicalis* was identified from cultural and microscopic features and confirmed by the Vitek 2 automated system. This result confirmed the invasive mycosis, obviously due to the underlying primary immunodeficiency, chronic granulomatous disease (CGD). Amphotericin was added, and he also received IV methylprednisolone for seven days. The patient improved and was weaned off oxygen with no fever. However, the patient was referred to a higher center for further workup, which confirmed CGD's diagnosis. He is on the list for HLA-identical bone marrow transplantation (BMT).

## 1. Introduction

Chronic granulomatous disease (CGD) is a rare inherited primary immunodeficiency disease caused by a defect in the nicotinamide adenine dinucleotide phosphate (NADPH) oxidase complex that leads to phagocytic dysfunction [1–3]. Impaired NADPH complex leads to phagocytic cells' inability to kill peroxidase-positive bacteria and fungi [1, 4]. Recurrent and slowly resolving pulmonary infections are the most common clinical manifestations of CGD in children [5–7]. The involvement of vital or large organs can contribute to morbidity and/or mortality in the affected patients [8].

The bacterial pathogens most frequently identified in CGD patients are *Staphylococcus aureus*, *Burkholderia cepacia*, *Serratia marcescens*, *Pseudomonas* species, *Nocardia* spp., *Salmonella* spp., *Escherichia coli*, and *Klebsiella* spp. [4]. Several reports have reported severe fatal candida pulmonary infections in patients with CGD [4, 5]. CGD patients are also susceptib to a wide range of fungal pathogens, namely *Aspergillus* spp., *Candida albicans.*Aspergillus [9, 10]. A study revealed that *Aspergillus* spp. was isolated from 78% of patients, *Candida* species from 32%, *Pneumocystis jirovecii* from 7%, and to a lesser extent. *Malassezia furfur, Fusarium* spp., mucormycosis, and *Penicillium chrysogenium*, 3.5% for each [11]. The mortality rate reported in this study was 35.7% [11]. PID patients are at high risk of developing fungal infections [11], indicating the need to determine the causative fungus, often by invasive diagnostics, to guide optimal and rational treatment [12]. This investigation aims to report a rare CGD case in an 11-year-old preadolescent boy with CGD and complicated candida pneumonia.

## 2. Case

On 21 November 2018, an 11-year-old preadolescent boy presented to our hospital with a productive cough, fever, and weight loss for four weeks. 1.5 months before the current illness, the patient was admitted to a local hospital with left neck lymphadenitis and underwent surgical drainage. The bacterial culture of the drained pus grew MRSA and received ten days of vancomycin and clindamycin. He was sent home in good condition. Symptoms started two weeks after discharge with a daily high-grade fever and productive cough. He had lost around 5 kg since his illness began with a poor appetite. There was no history of contact with tuberculous-infected patients. His older sister died six years ago due to severe pneumonia, and there was no clear underlying diagnosis. Parents are second-degree consanguineous with no family history of inherited lung disease.

**Figure 1:**
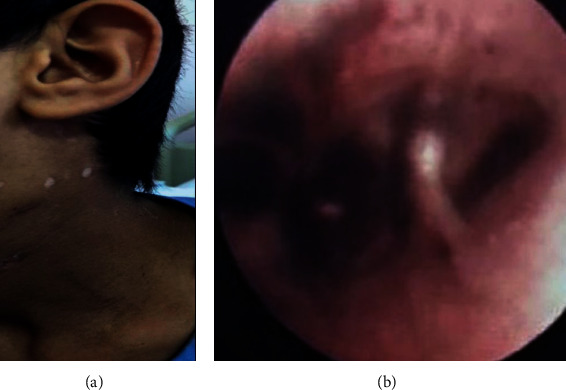
Left neck enlarged lymph nodes with healed scars (a) and flexible bronchoscopy (b) showing severe diffuse bronchitis with distal necrotic tissues.

**Figure 2:**
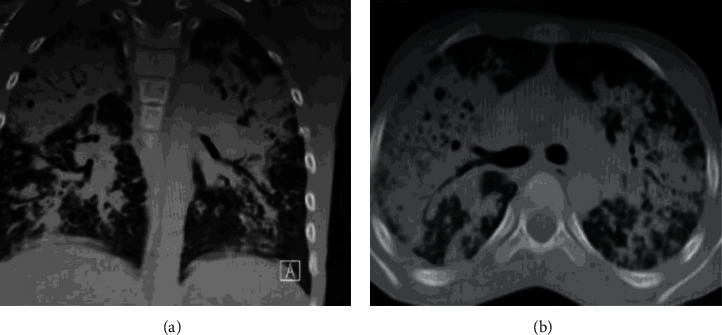
The chest CT scan showed patchy alveolar opacification (a) with multiple superior mediastinal lymph nodes (b).

On admission, he was dyspneic and febrile with an oral temperature of 39°C. His blood pressure was 110/60 mmHg, his heart rate was 120/min, his oxygen saturation was 85% on room air, and his respiratory rate was 20/min. Physical examination revealed left neck enlarged lymph nodes with healed scars (Figure 1). The chest revealed bilateral diminished air entry with bilateral coarse crackles. Peripheral blood leukocyte count was 28.260/*μ*L with 84% neutrophil, 11% lymphocyte, and 4.4% monocyte; hemoglobin and platelet counts were 9.5 mg/dL and 550 × 109 per liter. The erythrocyte sedimentation rate was 150 mm/h. All the results of biochemical tests were normal. A chest CT scan showed bilateral diffuse air space disease (Figure 2). A direct smear of sputum for acid-fast bacilli and tuberculous mycobacteria PCR was negative.

Upon admission (21 November 2018), the patient was started on broad-spectrum antibiotics linezolid, gentamycin, and meropenem. After five days of therapy, the fever continued with worsening chest X ray findings. So, the infectious team decided to start antituberculosis treatment empirically, given the rapid deterioration in his condition, which was later discontinued after getting the negative PCR and AFB smear results.

**Figure 3:**
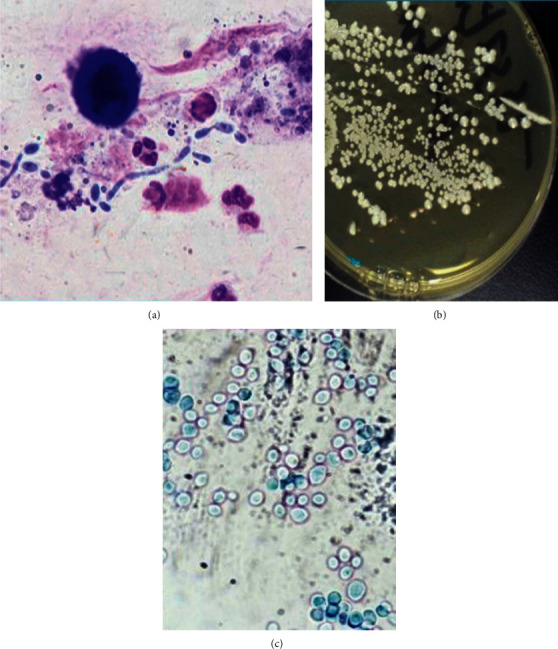
Demonstration of yeast in a direct smear made from sputum; note the presence of pseudohyphae and budding yeast cells (a). Growth of yeasts from sputum sample (strain mat532y) on SDA medium (b) and an indirect smear displaying yeast cells (LPCB ×100) (c).

On 29 November 2018, the demonstration of yeast in a smear made from sputum and the appearance of pseudohyphae and budding yeast cells (Figure 3(a)) dictated the need for fungal culture. Fungal cultures were performed on Sabouraud dextrose agar (SDA) and blood agar plates. Inoculated plates were incubated at 30°C and 37°C, respectively. Plates were examined daily for microbial growth. Initial identification of the grown organism was carried out using routine growth and colonial morphology criteria [13]-confirmation of identification of *Candida* spp. by the VITEK 2 system. The VITEK 2 automated system was used to confirm the identities of *Candida* species following protocols described by the manufacturer (bioMérieux Inc., Durham, NC 27712, USA). *Candida tropicalis* were isolated from a sputum specimen's culture (Figures 3(b) and 3(c)), which raised the possibility of invasive mycosis due to underlying primary immunodeficiency. Amphotericin was added at 1 mg/kg/day for two weeks, and he also received IV methylprednisolone (1 mg/kg/day twice daily for seven days), which was later tapered over two weeks. The patient improved and was weaned off oxygen with no fever.

On 15 December 2018, he was referred to higher centers for further workup. The bronchoscopy was done at the higher center, which showed diffuse bronchitis with necrotic involving mainstem bronchi (Figure 2). The patient's immune system was assessed. The ranges of the immunoglobulins (Ig) were normal, which include IgG 10.4 g/l (reference 7–16 g/l), IgA 2.81 g/l (reference 0.7–4 g/l), IgM 0.675 g/l (reference 0.4–2.3 g/l), and IgE 26.3 IU/ml (reference less than 200 IU/ml). The total protein level was 8.1 g/dl (reference 6.6–8.3 g/dl) and the albumin level was 2.4 g/dl (reference 3.5–5.2 g/dl). The oxidative burst measurement with flow cytometry using the dihydrorhodamine 123 assays was performed and confirmed the diagnosis of CGD through the comparison between control (reference range 98–100%) and the patient results (0.3%). The patient was started on daily prophylaxis withTrimethoprim-sulfamethoxazole (5 mg/kg/d, based upon the Trimethoprim) and itraconazole (5 mg/kg/day). The patient was then sent for HLA-identical bone marrow transplantation (BMT).

## 3. Discussion

Invasive fungal infections are the most important risk for patients with the chronic granulomatous disease (CGD) [14]. CGD is the most commonly encountered immunodeficiency syndrome affecting the phagocyte, which is indicated by repeated microbial infections and the formation of granulomas in tissue [15]. Investigations have reviewed the invasion of fungal infections in patients with CGD [10, 12, 16, 17]. These have indicated important optimal treatment strategies and guide research for improving outcomes [12].

The present CGD case is an example of a devastating fungal involvement. He presented with fever, hypoxia, and shortness of breath. The physical examination revealed enlarged lymph nodes in the neck and bilateral pulmonary involvement. It is an interesting encounter to isolate *Candida tropicalis* from the lung of this patient. *Candida* species are major causes of mucosal and invasive infections, leading to substantial morbidity and mortality [5, 18, 19]. Despite new antifungal drugs, mortality in patients with systemic candidiasis remains high [20]. Host-Candida interaction plays an important role in the effective elimination of the pathogen.

Due to the lack of well-controlled clinical trials to guide this condition's treatment, we follow the best available evidence of therapeutic strategies in treating CGD-related complicated fungal pneumonitis, which includes broad-spectrum antibiotics to treat coexistent bacterial infections, antifungal and anti-iflammatory agents (corticosteroid). These regimens have been used in various published case reports [6, 21–23]. The present case was successfully treated with a course of amphotericin B with added methylprednisolone (1 mg/kg/day twice daily for seven days), which was later tapered over two weeks. In CGD patients with complex and life-threatening infections, adding corticosteroids to the antifungal and antibacterial therapy is crucial. Several papers have recommended the use of corticosteroids as adjunctive therapy, notably, when vital organs are involved or there are obstructive symptoms [24–26].

Genetic studies have provided an important understanding of antifungal host defense and have identified possible targets for adjunctive therapy. In their article, the authors studied the genetic variations in the host defense against *Candida* and their implications for the treatment of mucosal and systemic candidiasis [27]. The immune system contains multiple components that protect specific groups of microorganisms. Chronic mucocutaneous candidiasis is an especially dramatic illustration of the role of the T-lymphocyte system in defense against opportunistic fungal infections, especially of the skin and mucous membranes. The most consistent defects involve the subnormal production of lymphokines by T-cells in response to *Candida* antigens [5].

Successful therapy of such fungal infection necessitates a combination of medicines, comprising antifungal drugs, such as clotrimazole, ketoconazole, or amphotericin B correction of the underlying immune defect with such agents as transfer factor [5, 28]. Amphotericin B, as a single agent in the treatment of systemic candidiasis, is a well-known approach, especially in neonates [5, 27]. In addition, the present case received IV methylprednisolone as an anti-inflammatory. Furthermore, the patient improved with no fever and did not need oxygen. Though the patient was treated for this severe and disseminated candidiasis, he was transferred to another hospital to proceed for HLA-identical bone marrow transplantation.

In conclusion, a severe invasive pulmonary fungal infection caused by *C. tropicalis* was diagnosed and successfully treated in an 11-year-old CGD preadolescent boy. The case substantiated the need for early diagnosis of CGD, which plays a significant role in patient's health outcomes. The underlying primary immunodeficiency (PID) disorders must be considered in any child with complicated pneumonia and inadequate response to broad-spectrum antibiotics. The family index case is the most important predictor of PID disorder diagnosis. Aggressive and early treatment of opportunistic fungal infections in CGD is critical to avoid end-organ damage until the patient gets BMT.

## Data Availability

The data used to support the findings of this study are available from the corresponding author upon request.
